# Exploring the use of Granger causality for the identification of chemical exposure based on physiological data

**DOI:** 10.3389/fnetp.2023.1106650

**Published:** 2023-03-15

**Authors:** S. Difrancesco, J. U. van Baardewijk, A. S. Cornelissen, C. Varon, R. C. Hendriks, A. M. Brouwer

**Affiliations:** ^1^ Department Systems Biology, The Netherlands Organisation for Applied Scientific Research (TNO), Leiden, Netherlands; ^2^ Department Human Performance, The Netherlands Organisation for Applied Scientific Research (TNO), Soesterberg, Netherlands; ^3^ Department CBRN Protection, The Netherlands Organisation for Applied Scientific Research (TNO), Rijswijk, Netherlands; ^4^ Circuits and Systems (CAS) Group, Delft University of Technology, Delft, Netherlands; ^5^ Centre for Research and Engineering in Space Technologies—CREST, Université Libre de Bruxelles, Brussels, Belgium

**Keywords:** Granger causality, chemical exposure, toxidrome detection, physiological data, support vector machine, machine learning

## Abstract

Wearable sensors offer new opportunities for the early detection and identification of toxic chemicals in situations where medical evaluation is not immediately possible. We previously found that continuously recorded physiology in guinea pigs can be used for early detection of exposure to an opioid (fentanyl) or a nerve agent (VX), as well as for differentiating between the two. Here, we investigated how exposure to these different chemicals affects the interactions between ECG and respiration parameters as determined by Granger causality (GC). Features reflecting such interactions may provide additional information and improve models differentiating between chemical agents. Traditional respiration and ECG features, as well as GC features, were extracted from data of 120 guinea pigs exposed to VX (*n* = 61) or fentanyl (*n* = 59). Data were divided in a training set (*n* = 99) and a test set (*n* = 21). Minimum Redundancy Maximum Relevance (mRMR) and Support Vector Machine (SVM) algorithms were used to, respectively, perform feature selection and train a model to discriminate between the two chemicals. We found that ECG and respiration parameters are Granger-related under healthy conditions, and that exposure to fentanyl and VX affected these relationships in different ways. SVM models discriminated between chemicals with accuracy of 95% or higher on the test set. GC features did not improve the classification compared to traditional features. Respiration features (i.e., peak inspiratory and expiratory flow) were the most important to discriminate between different chemical’s exposure. Our results indicate that it may be feasible to discriminate between chemical exposure when using traditional physiological respiration features from wearable sensors. Future research will examine whether GC features can contribute to robust detection and differentiation between chemicals when considering other factors, such as generalizing results across species.

## 1 Introduction

Wearable sensor technology is rapidly evolving, leading to higher quality data and more physiological parameters that can be monitored simultaneously. This development offers new opportunities to quickly detect and identify toxic chemicals based on their effects on the human body, enabling timely (self-administered) treatment when medical evaluation is not immediately possible, such as in the military battle field. Various specialized devices are under investigation for alcohol or substance abuse or are already commercially available, such as the Secure Continuous Remote Alcohol Monitor (SCRAM^®^) ankle monitor, which electrochemically detects transdermal alcohol (Davis-Martin et al., 2021). Even though direct chemical detection is the gold standard, it only allows for the monitoring of a limited number of compounds, making it unsuitable for ‘threat-agnostic’ monitoring. Furthermore, chemical detection is difficult for compounds that are toxic at extremely low systemic levels, such as is the case for novel synthetic opioids as carfentanil (Uddayasankar et al., 2018). Also note that differential diagnosis in the clinic is not always straightforward, as exemplified in the 2018 Salisbury poisoning incident, in which a nerve agent poisoning was mistaken for an opioid overdose (Eddleston & Chowdhury, 2020; Haslam et al., 2022). Indirect detection by measuring the compound’s (toxic) effects on the body (toxidrome) presents a promising approach for continuous, non-invasive monitoring of exposure to chemicals. Automatic algorithms may alert the possibly exposed individual or their colleague that quick countermeasures are required. In the battlefield such warnings could be especially helpful given that military personal likely ignore or suppress physical discomfort, and effects of chemicals are initially hidden for others by protective clothing and gas masks.

The effects of chemical intoxication on the body can be complex and multi-facetted. Machine learning models are suitable for complex pattern recognition analyses with relatively large numbers of parameters and previous studies showed that when applied to physiological data, they could detect various chemical intoxications. A study by Mahmud et al. (2018) employed various machine learning methods (decision tree, k-nearest neighbor, eXtreme Gradient Boosting) to detect opioid use based on data from a wrist-band with 99% accuracy. A study by Chang et al. (2021) showed that a neural network trained to recognize digoxin toxicity from electrocardiography (ECG) performed similarly to cardiologists and emergency room specialists, showing 84.6% sensitivity and 96.6% specificity. We previously showed that a machine learning model could accurately detect exposure to an opioid (fentanyl) or a nerve agent (VX) and differentiate between the two, based on continuously measured electroencephalography (EEG), ECG and respiration (whole-body plethysmography) data in guinea pigs (van Baardewijk et al., 2021).

While these studies successfully demonstrated the detection of chemical intoxication based on physiology, they all considered physiological parameters independently. However, under normal physiological conditions, the various biological systems of the body exhibit oscillatory patterns due to underlying feedback and feedforward mechanisms. For instance, heart rate is well-known to be regulated by many such mechanisms. In healthy people, successive beats do not occur at a constant rhythm, instead, (R-R) intervals show considerable variability. The largest contributor to heart rate variability (HRV) is respiratory sinus arrhythmia (RSA). The heart rate increases with inspiration and decreases with expiration, a mechanism by which the body optimizes pulmonary gas exchange (Hayano et al., 1996; Goldberger et al., 2013) and which is thought to be regulated mainly by central mechanisms (Gleb et al., 1936). These and other cardiorespiratory interactions vary under different circumstances, such as different breathing patterns (Stefanovska, 2002; Elstad et al., 2018; Lukarski et al., 2022). Various pathological conditions have been linked to changes in HRV, such as congestive heart failure, diabetes, and depression (Musialik-Łydka et al., 2003; Wang & Wang, 2011; Young & Benton, 2018; Hartmann et al., 2019). HRV has also been implicated as a useful marker for substance abuse (Koenig et al., 2015), withdrawal symptoms (Levin et al., 2019; Garland & Howard, 2021), and exposure to fine particulate matter (Riediker et al., 2018).

Even though the precise mechanisms of HRV remain poorly understood, these studies highlight the fact that the various physiological systems of our body do not function in an isolated manner. Instead, they coordinate and synchronize their functions to maintain a given physiological state. This holistic view of physiology is investigated in the field of Network Physiology (Bashan et al., 2012; Bartsch et al., 2015). Quantifying the interactions of physiological features under different (healthy and intoxicated) circumstances may improve the detection of toxic chemicals as well as increase our understanding of physiological mechanisms.

One method to quantify the (causal, i.e., time ordered) relationships between physiological features is Granger causality (GC), named after the econometrician who first described it in 1969 (Granger, 1969). This technique has been frequently applied in the financial sector, among others for investigating causal relationships between market factors, economic changes, stock prices, and stock price predictions (Výrost et al., 2015; Gao et al., 2018; Gherghina et al., 2020; Thakkar & Chaudhari, 2021). A variety of studies that used methods based on GC to quantify the interactions between physiological signals, mainly focused on heart rate, respiration, and arterial blood pressure. GC in these studies show how well the future of a physiological signal (e.g., heart rate variability) can be predicted from the present and the past of another signal (e.g., respiration) by means of linear vector autoregressive (VAR) models, and result in directionality and strength of interaction (Seth et al., 2015). A study by Faes et al. (2015) applied GC to map directional interactions in brain-brain and brain-heart networks in different sleep states, exemplifying the added value of GC in neuroscience (Porta & Faes, 2015; Seth et al., 2015). A study by Rozo et al. (2021) demonstrated that different methods to quantify RSA, based on GC principles, captured the cardiorespiratory changes expected during different non-REM sleep stages. Interactions between respiration, blood pressure and heart rate have been found to be influenced by factors such as body position (Mary et al., 2019) and deep versus normal breathing (Mary et al., 2018). These interactions have been used to make distinctions between healthy and diseased conditions under conditions such as congestive heart failure (Radovanović et al., 2018), and pre-eclampsia (Riedl et al., 2010). Recent studies successfully used GC between brain and heart signals to characterize epileptic seizures (Pernice et al., 2022) and GC between activity in different brain areas to distinguish between patients with cognitive impairment associated with epilepsy and healthy controls (Jiang et al., 2021).

Acknowledging the potential diagnostic value of physiological interactions in the context of exposure to toxics, and acknowledging GC as a way to quantify such interactions, we here determine the GC interactions within and between both respiration and ECG parameters under healthy and intoxicated (fentanyl or VX) conditions. RSA, that we already discussed above, is the most studied form of cardiorespiratory GC interactions, despite the identification of other forms (cardiorespiratory phase synchronization: Bartsch et al. (2007), and time delay stability; Bartsch et al., 2014). One of the reasons for this is that RSA can be directly estimated using predictability and casual measures based on GC, applied to the raw respiratory signal and the tachogram, derived from the ECG. Such measures are often used to estimate the information transferred from a driver, often the respiration, to a target signal (e.g., the tachogram). Here, however, we study the cardiorespiratory interactions not by predicting one (close to) raw signal from the other, but by looking at the effect that specific respiratory higher order features have on the morphological and rhythm features of the ECG, such as the effect of peak inspiratory flow on the interval between successive heart beats, and *vice versa*. Interactions between these features are also examined within modality. Relying on higher order features is important from the perspective of our envisioned ultimate application of using wearables for diagnostics in the field, where the quality of the raw physiological signals and their synchronization is likely compromised. Since it is currently unknown how exposure to fentanyl and VX affect GC interactions, we first provide an overview of the interactions for each condition. Next, we evaluate the contribution of traditional ECG and respiration features as well as GC features in machine learning models that aim to differentiate between exposure to fentanyl and VX over 45 min following exposure as well as over the first 15 min of exposure (which we considered as a cut-off for a timely treatment in an exposure scenario). The study here is an updated and extended version of a previous proceedings paper [van Baardewijk et al. (2022)].

## 2 Materials and methods

### 2.1 Sample

Data comprised four existing physiological datasets of freely moving guinea pigs, exposed to VX (*n =* 62) or fentanyl (*n =* 71). The animal procedures were as described previously (Joosen et al., 2017). Briefly, VX was obtained from the in-house synthesized TNO stocks. Purity upon issue was >98%. Fentanyl citrate (European Pharmacopoea grade) was purchased from Spruyt-Hillen (IJsselstein, Netherlands). Purity was >99%. VX was either dissolved in 2-propanol (IPA) to the required concentration or applied as neat agent. The VX doses applied were 1–2 mg/kg dermally, corresponding to approximately 1.5–3 times the 24 h LD50 values in guinea pigs (Rice et al., 2015). The fentanyl doses ranged from 0.05 to 8 mg/kg (intravenous bolus) and 0.4–32 mg/kg (subcutaneous), selected to elicit varying degrees of respiratory depression. Fentanyl was dissolved in phosphate-buffered saline (PBS) to the required concentration before administration. For continuous measurements, animals were surgically equipped with ECG leads. Two leads were sutured in the superficial muscles under the skin right below the right collar bone and between the second and third rib (configuration II). ECG data were transmitted wirelessly to a hardware system (Data Sciences International (DSI), St. Paul, MN, United States) using F40-EET (nominal sampling rate 240 Hz) or HD-S02 (nominal sampling rate 375 Hz) telemetry devices. Unrestrained respiratory plethysmography (URP) data were obtained using whole-body plethysmography cages, connected to a Universal XE signal conditioner (DSI). Telemetry and plethysmography data were upsampled simultaneously at 1,000 Hz using the Ponemah Physiology Platform (v5.41) software, in order to combine the modalities into a single dataset. Under typical conditions, the synchronization error of the two modalities was within 150 ms. For each animal, at least 30 min of data were acquired before exposure. The final sample included 120 animals; nine animals were excluded because they belonged to a placebo group, four animals were excluded because they died during the experiment.

### 2.2 Preprocessing: From raw data to the extraction of ECG and respiratory features

**FIGURE 1:**
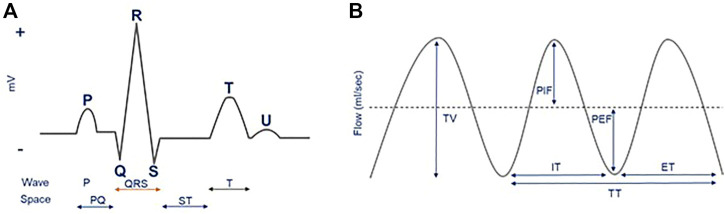
ECG **(A)** and respiration **(B)** features illustrated in schematic raw ECG and respiratory signals. In the current study, we used R-R interval (RR-I), ST elevation (ST-E), R height (R-H) and QRS duration (QRS) as traditional ECG features. For respiration, the traditional features that were used were tidal volume (TV), peak inspiratory flow (PIF), peak expiratory flow (PEF), inspiratory time (IT), expiratory time (ET), and total time (TT).

Physiological data were preprocessed using Ponemah^®^ Software. The signals were inspected visually to identify and exclude artifacts related to movements and sudden ambient pressure changes. All derived features were subsequently exported in a beat-to-beat format for further processing. The following four traditional ECG features were extracted from ECG data: R-R interval (RR-I), ST elevation (ST-E), R height (R-H) and QRS duration (QRS). For respiration, the six traditional features that were extracted from URP data were: tidal volume (TV), peak inspiratory flow (PIF), peak expiratory flow (PEF), inspiratory time (IT), expiratory time (ET), and total time (TT; TT = IT + ET). These ECG and respiratory features are illustrated in Figures 1A, B, respectively.

To identify and remove signal artifacts, *z-*scores were determined for 20 s moving windows (shifted in steps of 1 s). Datapoints with a *z-*score higher than 3 or lower than −3 were removed.

Data around the moment of exposure (from 5 min before to 5 min after) were excluded to prevent any handling effects related to administering the chemical potentially influencing the data.

### 2.3 Traditional ECG and respiratory features

Extracted features were aggregated over successive 5-min windows for data from 30 min before exposure to 45 min after exposure. Within-animal centering of features was done by subtracting the baseline, which was defined as the average feature value as recorded during the first 15 min (i.e., from 45 min until 30 min before exposure). Missing data were linearly interpolated. Such features were used as input for Support Vector Machine (SVM) classification analysis (Cortes & Vapnik, 1995) as described later.

### 2.4 Granger causality (GC) features

In this study, a bivariate formulation of GC was used. In such a formulation a system consists of two variables X and Y. Y causes X, in the Granger sense, if the past of Y ( $Y_{n}^{l}$ ) provides information about the future of variable X, given the past of X ( $X_{n}^{k}$ ), where $Y_{n}$ and $X_{n}$ denote the present value of Y and X respectively and:

$$X_{n}^{k}=\left[X_{n-1},X,\ldots ,X_{n-k+1}\right],$$ (1)

$$Y_{n}^{l}=\left[Y_{n-1},Y_{n-2},\ldots ,Y_{n-l+1}\right],$$ (2)

where k and l represent the time lags.

For extracting GC features between each pair of features, the different time series (i.e., extracted features) were resampled at aligned points in time. For subsequent windows of 100 ms, data points in a specific window were averaged. Average data points were linearly interpolated and data were resampled at 10 Hz. The time series were then detrended by means of differencing.

GC features were determined for each animal, each 5-min window, and each of the 90 combinations of traditional ECG and respiratory features (four ECG and six respiratory features as described in 2.2 gives a total of 10 features; leading to 10*10–10 = 90 unique combinations). To determine the optimal GC lag, the Vector Autoregression (VAR) on the healthy data of all animals was calculated by varying the lag from 1 to 50 100 ms-windows. The optimal lag value was the one with the lowest average Akaike information criterion (AIC) (Akaike, 1974). In this case, the optimal lag was found to be 35 100 ms-windows, i.e. 3.5 s.

Statistical significance of GC was determined with an SSR F-test, resulting in a *p*-value (*α* = 0.05) for each combination in each 5-min window. F-statistic values were used as GC features in the prediction models. Percentages of statistically significant GC features were plotted to give an impression of which interactions between ECG and respiratory features were more present than others in the current sample.

To extract Granger causality features and to identify the optimal lag as just described, we used Python and the statsmodels module version 0.12.2. Specifically, the “VAR” class from the statsmodels module was used to calculate the VAR. For each lag a fit was done and from the result the AIC value was used. The function “grangercausalitytests” from the statsmodels module was used for calculation of the Granger Causality. GC for each pair of features was calculated by putting the values of the two features in a two-column dataframe and using that as the first input for this function. A list of one element was used for the “maxlag” parameter, with the optimal lag as the only element, so only this lag was used as parameter for the test. From the output of this function, the *p*-value from “ssr_ftest” was used for statistical significance as described above.

### 2.5 Feature selection, classification of exposure and classification evaluation

Using classification analysis we explored whether GC could support classification of respiration and ECG data into either exposure to VX or fentanyl. Twenty percent of the data (21 of the animals) was set aside as a test set to evaluate the final model after the training phase (using data of 99 animals). The proportion of animals exposed to either VX and fentanyl was held constant between the training and the test set.

A standard scaler was used to standardize the data before using the SVM. After that, feature selection, classification and evaluation of the classification was performed in the dataset stratified by time from the exposure (i.e., 5–45 min and 5–15 min from exposure).

Because the final training set was composed of 100 features (4 traditional ECG features, 6 traditional respiratory features, and 90 GC features), feature selection was performed. The minimum Redundancy Maximum Relevance (mRMR) algorithm was used to rank features by their importance. mRMR ranks features high if they are mutually far away from each other (i.e., minimum redundancy) while still correlating strongly with the to be classified variable (i.e., maximum relevance) (Peng et al., 2005). After that, an SVM was used to classify the exposure to the type of chemical based on varying numbers of features. Specifically, an SVM (with standard hyperparameters) was trained by adding one of the ranked features at the time, following their rank and starting with the first. Leave-one-group-out (LOGO) cross validation was applied to evaluate the accuracy of the classification model by leaving one animal out at each iteration on the training set. The average accuracy varying by the number of ranked features was plotted to qualitatively select the optimal number of features. A similar approach has been used before (Peng et al., 2005). The top traditional as well as GC features were selected.

A grid-search was then used for tuning the SVM and optimize the following hyperparameters: kernel, C or “regularization parameter”, and gamma. The kernel defines whether the decision boundary is linear or not. Here, a linear and a radial basis function were used as candidate kernels. The constant C represents the tradeoff between minimizing the training set error and maximizing the margin. Gamma is a parameter for nonlinear kernels; gamma controls the influence of each feature on the decision boundary. C and gamma were initialized on different scales ranging from 0.0001 to 100. LOGO was used in this step to optimize the SVM as well as performing internal validation for the best set of parameters. Finally, the tuned SVM was evaluated on the test set (i.e., external validation).

Python 3.9 was used to perform the analyses. The pymrmr library was used to rank features and the sklearn library was used to build the classification model.

## 3 Results

**FIGURE 2:**
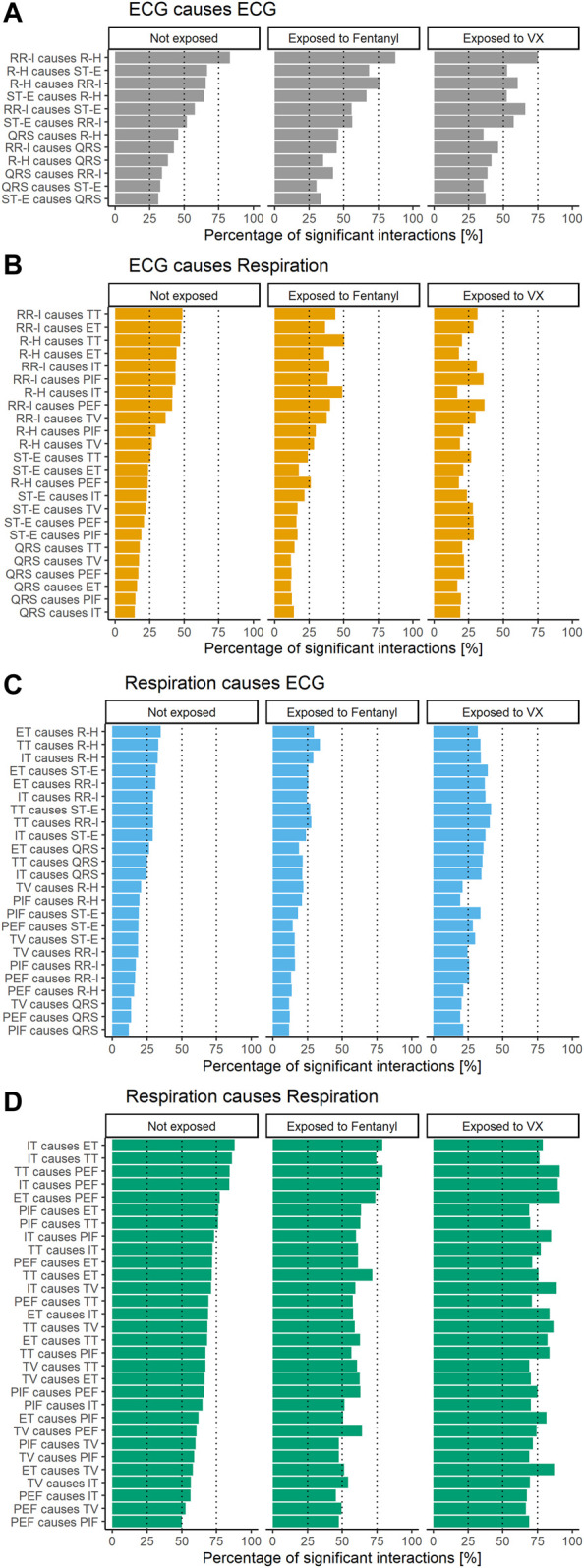
Percentage of 5-minute windows with significant GC per condition and feature combination for ECG causing ECG **(A)**, ECG causing respiration **(B)**, respiration causing ECG **(C)**, and respiration causing respiration **(D)**.

Figure 2 shows the percentage of 5-min windows with significant GC. Not exposed conditions are shown on the left, exposure to fentanyl in the middle and exposure to VX on the right. Results are shown separately for ECG causing ECG features a), ECG causing respiration features b), respiration causing ECG features c) and respiration causing respiration features d). All feature combinations were GC related for more than 25% of the 5-min windows within modality (ECG causing ECG and respiration causing respiration features). Most feature combinations were GC related for more than 20% of the cases between modalities (ECG causing respiration and respiration causing ECG features). Patterns appear to differ between the three exposure conditions.

**FIGURE 3:**
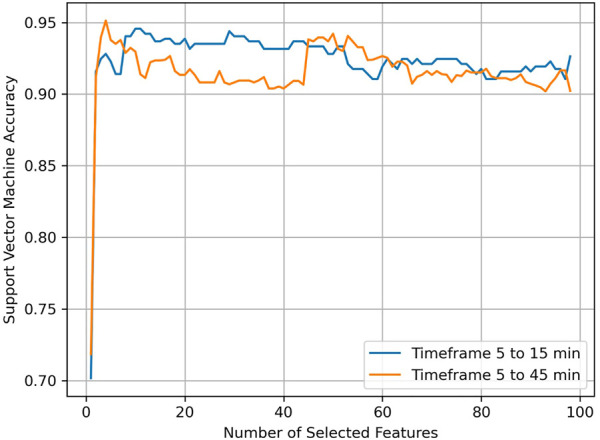
SVM accuracy as a function of number of selected features.

The feature selection step showed that with only the top three features (ranked PEF, TT, PIF for the first 45 min; ranked PEF, PIF, ET for the first 15 min) the accuracy of the SVM on the training set already reached a high accuracy for both the 45 and the 15 min case (Figure 3). The following top three features in the feature selection step were all GC features. For the first 45 min from exposure, these were (ranked) IT causes ET, RR-I causes R-H, PIF causes TV. For the first 15 min from exposure, these were (ranked) RR-I causes R-H, TT causes PEF, PIF causes TV.

**FIGURE 4:**
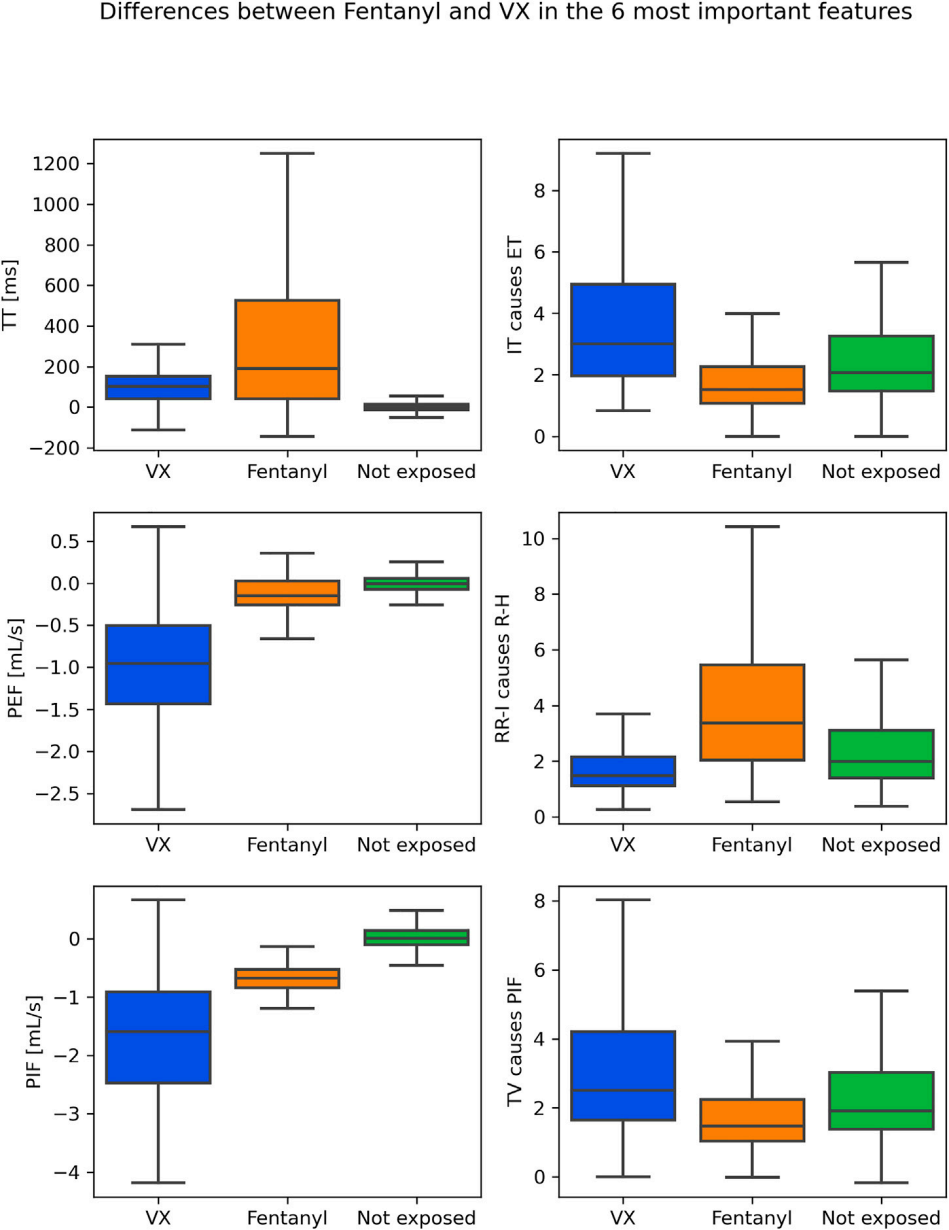
Boxplots for VX (blue) and fentanyl (orange) exposure in the most important features in the first 45 min from exposure*:* top 3 traditional features on the left, top 3 GC features on the right. For reference, values for the not exposed condition are shown as well (green).

**FIGURE 5:**
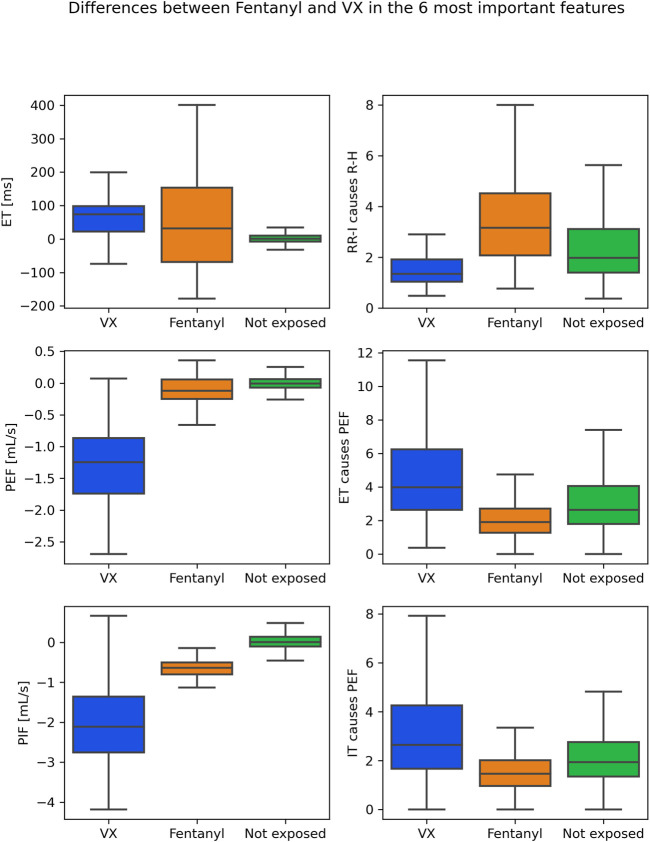
Boxplots for VX (blue) and fentanyl (orange) exposure in the most important features in the first 15 min from exposure*:* top 3 traditional features on the left, top 3 GC features on the right. For reference, values for the not exposed condition are shown as well (green).

Figures 4, 5 provide insight into how the features that came out as the most relevant features for differentiating between fentanyl and VX differed between these chemical exposure conditions. The left panels in Figure 4 show how the top three (traditional) variables differed for the first 45 min after exposure. TT, PEF and PIF were all lower for fentanyl than for VX. The left panels in Figure 5 show how the three highest ranked features (PEF, PIF and ET) differed between the chemical exposure conditions when only the first 15 min from exposure were considered. As for the 45 min, PEF and PIF were lower for fentanyl than for VX. ET was higher for fentanyl than for VX. The right panels in Figures 4, 5 indicate how the top GC features differed between conditions. The GC for ECG features (RR-I causes R-H in both the 45- and 15 min case) was low in VX compared to fentanyl. In contrast, the GC for respiration features (with slightly different GC combination popping up for the 45- and 15-min case) was low in fentanyl compared to VX. To provide insight as to how the exposure conditions compare to the not exposed condition, Figures 4, 5 include these data as well.

Table 1 shows optimal hyper parameter settings used in the SVM. Table 2 shows internal validation and external validation results. Accuracy of the SVM using traditional features alone was high for both the first 45 min and the first 15 min (respectively 95% and 97% for the test sets). Results were comparable when using traditional features alone versus traditional and GC features in both the 45 and 15 min time frames, indicating that GC features did not add to traditional features. Still, models using only GC features had an accuracy of 79%, confirming the impression from Figure 2 that relations between physiological features differed between exposure to VX and fentanyl.

**TABLE 1:** SVM hyperparameters tuning.

	Kernel	C	ɣ
5 to 45 min from exposure			
Traditional features^a^	rbf	90	0.02
GC features^b^	linear	0.09	5
Traditional + GC features^a^ ^+^ ^b^	rbf	90	0.01
5 to 15 min from exposure			
Traditional features^c^	rbf	100	0.10
GC features^d^	rbf	1.50	0.50
Traditional + GC features^c^ ^+^ ^d^	rbf	100	0.10

^a^ Features = TT, PEF, PIF.

^b^ Features = IT causes ET, RR-I causes RH, TV causes PIF.

^c^ Features = ET, PEF, PIF.

^d^ Features = RR-I causes R-H, ET causes PEF, IT causes PEF.

**TABLE 2:** Internal and external validation of SVM prediction of chemical exposure.

	Internal validation	External validation
	Accuracy, mean (SD)	Accuracy
5 to 45 min from exposure		
Traditional features^a^	0.954 (0.215)	0.950
GC features^b^	0.754 (0.425)	0.790
Traditional + GC features^a^ ^+^ ^b^	0.953 (0.203)	0.940
5 to 15 min from exposure		
Traditional features^c^	0.970 (0.260)	0.970
GC features^d^	0.767 (0.614)	0.790
Traditional + GC features^c^ ^+^ ^d^	0.958 (0.256)	0.970

^a^ Features = TT, PEF, PIF.

^b^ Features = IT causes ET, RR-I causes RH, TV causes PIF.

^c^ Features = ET, PEF, PIF.

^d^ Features = RR-I causes R-H, ET causes PEF, IT causes PEF.

## 4 Discussion

We explored the relation between different ECG and respiration parameters under healthy conditions, after exposure to fentanyl, and after exposure to VX using Granger causality. We were especially interested in whether the interactions between physiological signals, quantified using Granger’s method, could be used to improve discrimination by machine learning models between exposure to VX and fentanyl relative to using traditional features alone. Quantification of (cardiorespiratory) interactions may be useful in improving machine learning models designed to detect acute chemical intoxication and discriminate between chemicals based on non-invasive physiological data, as well as improve our understanding of chemicals’ toxic effects.

SVM classification showed that it was already possible to discriminate between VX and fentanyl with high accuracy (95%) after 15 min, using traditional features. While models using GC features alone showed that these features contained information, adding them did not result in improved classification of the already high accuracy reached by using only traditional features. Respiration features were the most important to discriminate between the two types of exposure. This is consistent with the different pharmacological mechanisms by which both compound classes exert their toxic effects and the used administration routes. Opioids, such as fentanyl, directly bind to the mu opioid receptor (MOR), disrupting the central respiratory drive, controlled by various respiratory centers in the brainstem (Pattinson, 2008; van der Schier et al., 2014). In the current data sets, fentanyl poisoning occurred *via* intravenous and subcutaneous exposure, leading to rapid intoxication. Nerve agents, such as VX, cause a cholinergic crisis, leading to a wide palette of signs and symptoms typical for nicotinic and muscarinic overstimulation. In the current data sets, VX poisoning occurred *via* dermal exposure, resulting in a steady progression of toxicity, where bradycardia and hypothermia are typically observed first and respiratory distress at a more severe state (Hamilton et al., 2004; Mumford et al., 2008). Our finding that it was already possible to discriminate between the chemicals in the first 15 min from the exposure with good accuracy demonstrate the feasibility to discriminate between chemical exposure when using respiration data that may be measured using wearable sensors. This is important to provide timely interventions to reverse the effects of chemicals’ exposure. It remains to be seen how the accuracy of models such as these varies with other compounds and other dosages.

The fact that classification accuracy was already high when using traditional features alone made it difficult for GC features to further improve that. Models based on GC features alone showed that these features contained information, but they performed poorly compared to models based on traditional features alone. This may be explained by two main limitations of this work. Firstly, the GC features were computed under the assumptions of stationarity and joint Gaussian distribution. As a result, only the linear interactions could be captured, thereby ignoring possible nonlinearities that could be strongly affected by the exposure. Therefore, future work should focus on the quantification of these possibly nonlinear interactions (Rozo et al., 2021). Secondly, the interactions between the features were assumed to be constant throughout the 15 or 45 min after the exposure. It is, however, still unknown whether such interactions change towards the general health deterioration caused by the exposure. Future studies will investigate if such dynamic changes are stronger and occur faster in the cross-modality features when compared to traditional ECG and respiratory features.

While the current work did not demonstrate a large contribution of Granger causality features for the purpose of distinguishing between exposure to different toxic chemicals, these features may add value for the purpose of generalizing results across species and across movement conditions. Automatic and early detection of exposure to toxic chemicals can save human lives, but studying the physiological effects of these chemicals can only be done in animals where it is questionable how well these models generalize to humans. Also, large variations in body movement and posture may make it hard to automatically detect and exposure to chemicals. In future work we hope to examine how traditional as well as interaction features vary across species, movement and exposure conditions in order to select the features that are insensitive to variations in species and movement. As mentioned before, the cardiorespiratory interactions can be analyzed from the GC perspective using the raw respiratory signal and the tachogram. This study, instead, highlights that the GC relations between respiration and ECG are also prominent when higher order variables are used, which are derived from the raw signal (e.g., RR-I, TT), suggesting that exact synchronization and high-quality raw signals may not be essential. This is an advantage since such high-quality signals are known to be difficult to record using wearables and under ambulatory conditions. Future work will examine how traditional GC relations between respiration and ECG compare for these different approaches, and for data from wearables compared to high-end equipment.

## Data Availability

The original contributions presented in the study are included in the article/supplementary materials, further inquiries can be directed to the corresponding author.
